# Comparative Study of Field-Effect Transistors Based on Graphene Oxide and CVD Graphene in Highly Sensitive NT-proBNP Aptasensors

**DOI:** 10.3390/bios14050215

**Published:** 2024-04-26

**Authors:** Anastasiia Kudriavtseva, Stefan Jarić, Nikita Nekrasov, Alexey V. Orlov, Ivana Gadjanski, Ivan Bobrinetskiy, Petr I. Nikitin, Nikola Knežević

**Affiliations:** 1 Center for Probe Microscopy and Nanotechnology, National Research University of Electronic Technology, Zelenograd, Moscow 124498, Russia; 2 BioSense Institute—Research and Development Institute for Information Technologies in Biosystems, University of Novi Sad, 21000 Novi Sad, Serbia; sjaric@biosense.rs (S.J.); igadjanski@biosense.rs (I.G.);; 3 Prokhorov General Physics Institute of the Russian Academy of Sciences, Moscow 119991, Russia

**Keywords:** graphene, graphene oxide, heart failure, field-effect transistor, point-of-care diagnostic, aptamer

## Abstract

Graphene-based materials are actively being investigated as sensing elements for the detection of different analytes. Both graphene grown by chemical vapor deposition (CVD) and graphene oxide (GO) produced by the modified Hummers’ method are actively used in the development of biosensors. The production costs of CVD graphene- and GO-based sensors are similar; however, the question remains regarding the most efficient graphene-based material for the construction of point-of-care diagnostic devices. To this end, in this work, we compare CVD graphene aptasensors with the aptasensors based on reduced GO (rGO) for their capabilities in the detection of NT-proBNP, which serves as the gold standard biomarker for heart failure. Both types of aptasensors were developed using commercial gold interdigitated electrodes (IDEs) with either CVD graphene or GO formed on top as a channel of liquid-gated field-effect transistor (FET), yielding GFET and rGO-FET sensors, respectively. The functional properties of the two types of aptasensors were compared. Both demonstrate good dynamic range from 10 fg/mL to 100 pg/mL. The limit of detection for NT-proBNP in artificial saliva was 100 fg/mL and 1 pg/mL for rGO-FET- and GFET-based aptasensors, respectively. While CVD GFET demonstrates less variations in parameters, higher sensitivity was demonstrated by the rGO-FET due to its higher roughness and larger bandgap. The demonstrated low cost and scalability of technology for both types of graphene-based aptasensors may be applicable for the development of different graphene-based biosensors for rapid, stable, on-site, and highly sensitive detection of diverse biochemical markers.

## 1. Introduction

Carbon-based nanomaterials are actively exploited in the development of biosensors due to several unique properties, like their high stability, inertness, biocompatibility, high sensitivity, and relatively easy functionalization. Graphene and its derivatives possess high potential as materials for point-of-care (POC) diagnostics due to the well-developed scalable processes of graphene production, either based on chemical vapor deposition (CVD) roll-to-roll technology [1], or ink-based methods for graphene oxide (GO) deposition [2]. Even though the production costs for the CVD graphene and GO-based biosensors are similar, the properties and applications’ functionality differ between these two types of graphene-based biosensors. Namely, CVD graphene represents an almost ideal intact crystal with zero band gap [3], while GO is a semiconducting material that preserves a band gap even after reduction [4]. However, regardless of these differences, both types of named graphene-based biosensors can be applied for detection of target molecules and molecular moieties in the analyte.

One of the promising graphene-based configurations of low-cost biosensors is the field-effect transistor (FET) channel modified with a target-specific aptamer [5,6]. Aptamers have attracted significant attention lately as receptors in biosensors due to a relatively low-cost manufacturing process and higher stability when compared to, e.g., antibodies [7]. The mechanism of graphene-based aptasensors is based on electrostatic gating, where there is a modulation of distance or the number of charges between the graphene surface and the analyte and/or aptamer. This approach has been demonstrated to work perfectly for various analytes like ions, small molecules, proteins, and even bacteria [8]. The simple and reliable structure of the sensing layer and straightforward signal transducing of biochemical reaction to electrical data makes FET biosensors ideal candidates for real-time and POC applications (e.g., population screening during outbreaks of infectious diseases). However, one of the downsides of these biosensors, as shown recently in a thorough survey of GFET-based aptasensors using the data of more than 5000 measurements [9], is that the variation in sensor’s response can be more than 300%, even for devices produced in one batch. The response variation is less noticeable when using blank solutions and greatly increases when measuring target analyte. Moreover, when the charges from the analyte are similar and interfere with the charges from aptamers, e.g., when targeting small peptide molecules, achieving a reliable biosensors performance becomes very challenging [10].

B-type natriuretic peptide (BNP) and its biologically inactive N-terminal proBNP form (NT-proBNP) [11] have an important role in regulating circulation by acting on blood vessels in response to wall stress. Ventricular cardiomyocytes are the main regulators of heart contractions and contain NT-proBNP. When heart muscles are damaged, the NT-proBNP is released to the blood stream and can be found in other bodily fluids as well, such as saliva. NT-proBNP is considered a promising heart failure (HF) marker for POC diagnostic applications [12,13]. Moreover, recent efforts have been focused on non-invasive methods of NT-proBNP measurements where rapid but still accurate testing is required, for example, in the diagnosis of acute HF for patients in intensive care unit admission who already have an increased mortality risk [14,15]. The most promising approach to measuring this biomarker in such conditions is to monitor its concentration in the saliva of patients. The advantages of using saliva for biological sampling include its fast and easy collection methods without the need for complex and invasive preparations. Nevertheless, detection from saliva is still challenging as the concentration of NT-proBNP in saliva can be lower than 1 pg/mL [14]. The high sensitivity of FET-based biosensors can allow for even the lowest concentration of the biomarker in saliva to be measured, even though an additional challenge is the fact that the size of NT-proBNP is comparable to the specific aptamer [16,17], adding to the complexity of signal interpretation when measuring with an FET-based aptasensor. To this aim, it is important to suggest that aptasensors technology will decrease the uncertainty in measurements of analyte trace levels.

In this work, we discuss cost-effective technology for FET-based biosensors using commercial interdigitated electrodes (IDEs) in contact with the graphene channel. A novel graphene FET (GFET) based on CVD graphene transferred on IDEs was constructed with its surface functionalized with the aptamer specific for NT-proBNP. The performance of the developed CVD GFET-based biosensor was compared with a reduced GO-FET (rGO-FET) biosensor that was recently developed by our group, and the initial results were published elsewhere [10]. Both devices were used to measure a wide range of NT-proBNP concentrations from 1 fg/mL to 10 ng/mL in both buffer solution and dissolved artificial saliva (AS). AS is considered a good model to study possible interferences on the sensors’ performance in bodily fluids [18]. Both sensors demonstrated high sensitivity to the analyte; however, a reliable limit of detection (LoD) as low as 100 fg/mL in dissolved AS corresponding to sensitivity of 1 pg/mL in saliva was observed only for rGO-FET. The characteristics of the two types of graphene-based biosensors are discussed in terms of the observed differences and perspectives of the two technologies for aptasensor application. For the first time, we demonstrate that for sensing of small peptide molecules, like NT-proBNP, whose size is comparable to the size of the aptamer, the increase in thickness of the electrical double layer (EDL) due to the higher roughness of the sensing surface and the presence of a bandgap can provide higher dynamic range and lower LOD, and thus, better performance for rGO-FET biosensors.

## 2. Materials and Methods

### 2.1. Materials

Graphene monolayer on Cu foil was purchased from Graphenea (San Sebastian, Spain). Poly(methyl methacrylate) (PMMA, average Mw ~350,000, powder), 2-propanol, toluene and N-methyl-2-pyrrolidone (NMP), (3-aminopropyl) triethoxysilane (APTES), and cysteamine were obtained from Sigma-Aldrich (USA). Tetrahydrofuran (THF), 99.9% was purchased from Carl Roth (Karlsruhe, Germany). Acetone was purchased from Honeywell (Charlotte, NC, USA). A 2 mg/mL GO in suspension (Sigma Aldrich (St. Louis, MA, USA), catalog number 763705) is presented in the form of a monolayer sheet with a size of less than 10 µm. Pyrenebutyric acid N-hydroxysuccinimide ester (PBASE), ethanolamine (ETA), and bovine serum albumin lyophilized powder (BSA) were obtained from Sigma Aldrich (St. Louis, MA, USA). Dimethylformamide (DMF) and isopropyl alcohol (IPA) were purchased from Component-Reactive (Moscow, Russia) and Sigma Aldrich (St. Louis, MA, USA).

Tablets of phosphate buffer (PBS, 2.7 mM KCl, 137 mM NaCl, 10 mM phosphate) and Tween were purchased from Sigma-Aldrich (USA). The N20a aptamer, modified with a 5′-amine (5′-NH2-GGCAGGAAGACAAACAGGTCGTAGTGGAAACTGTCCACCGTAGACCGGTTATCTAGTGGTCTGTGGTGCTGT-3′) [16,19], was purchased from DNA Synthesis, LLC (Moscow, Russia). It was purified using denaturing polyacrylamide gel electrophoresis (PAGE) to achieve a mass of 3.1 nM (69.5 µg). NT-proBNP 1.18 mg/mL frozen solutions in 10 mM potassium phosphate, pH 7.4, 150 mM NaCl, 0.7 mg/mL of cardiac troponin I (cTnI), and 1.18 mg/mL of proBNP in 10 mM HCl were purchased from HyTest Ltd. (Turku, Finland).

### 2.2. Fabrication of the GFET and rGO-FET Devices

Two types of FETs were investigated: GO monolayer ink and CVD monolayer graphene transferred on commercially available IDEs G-IDE222 (Drop Sens, Oviedo, Spain).

The rGO-FET device was produced by the method described in our previous work [10]. In brief, graphene oxide was diluted in a water/NMP mixture (10/90%) to 0.2 mg/mL. The GO suspension was drop-casted on gold electrodes modified with cysteamine and incubated for 2 h. Glass between the electrodes was activated by APTES to enhance the adhesion of GO monolayers. GO was reduced using hydrazine vapor at 80 °C for 2 h, followed by thermal annealing at 200 °C for 1 h.

Monolayer graphene on copper foil (30 μm thickness) was transferred onto the working electrodes using the wet transferring method with PMMA support. In short, PMMA was spin-coated over the graphene/Cu flake and pre-baked at 60 °C for 5 min. The Cu foil etching was initiated in an aqueous mixture of hydrochloric acid, hydrogen peroxide, and water (1:2:20 *v*/*v*) for 3 min and then transferred into 0.1 M ammonium persulfate aqueous solution to etch the Cu foil completely for 2 h. After several water baths to wash away the etchant, the graphene/PMMA flake was transferred onto the electrode and left overnight to dry. PMMA was cleaned with boiled acetone (three baths) and a boiled THF/water mixture (1:3 *v*/*v*) to completely dissolve and remove the PMMA film, then thoroughly rinsed with water and dried with an N_2_ gun.

### 2.3. Assembling of the GFET and rGO-FET Aptasensors

The FET-based aptasensors were assembled in a similar way as previously described for graphene devices [5,10]. PBASE linker was immobilized on the FET channel by a 3 h incubation of a 5 mM PBASE in DMF under −0.3 V potential applied to an auxiliary electrode [20]. The FET channel was then rinsed consequently with DMF, IPA, and DI water to remove reagent excess. A 100 µL drop of 100 nM of N20a aptamer in 1×PBS solution (with pH = 7.4) was introduced into the well mounted on a FET channel and kept overnight in humid atmosphere to ensure aptamer binding to the PBASE linker. After rinsing several times in PBS solution, 100 mM ethanolamine solution in PBS was introduced and kept for an hour in the well to block and deactivate non-bonded reactive groups. To block remaining channel surface, a 0.5% BSA aqueous solution was incubated on FET IDE chip for 30 min.

### 2.4. FET Devices Characterization

Atomic force microscopy (Solver-PRO, NT-MDT, Moscow, Russia) was used to estimate the surface roughness of the graphene channel. The quality of FET channels was investigated by microRaman spectroscopy (Centaur HR, Nanoscan Technology, Dolgoprudny, Moscow region, Russia) with a 100× objective (NA = 0.9) at a 532 nm wavelength with a laser power of 0.5 mW. Current voltage characteristics (CVC) were measured using Ag/AgCl (Science Products GmbH) as a liquid gate electrode with a semiconductor parameter analyzer IPPP1/5 (MNIPI, Minsk, Belarus) connected to the source and drain electrodes.

The NT-proBNP was dissolved in 0.1×PBS with the addition of Tween (0.01%) to avoid sticking of proteins to the tube wall. Solutions of biomarker were prepared of different concentrations from 1 fg/mL to 10 ng/mL [21]. Artificial saliva (AS) was ordered from “Apoteka Beograd” (Belgrade, Serbia) and dissolved 10 times in 0.01×PBS. NT-proBNP was dissolved in AS the same way as described above for PBS.

## 3. Results and Discussion

### 3.1. GFET vs. GOFET Channel Surface Analysis

**Figure 1:**
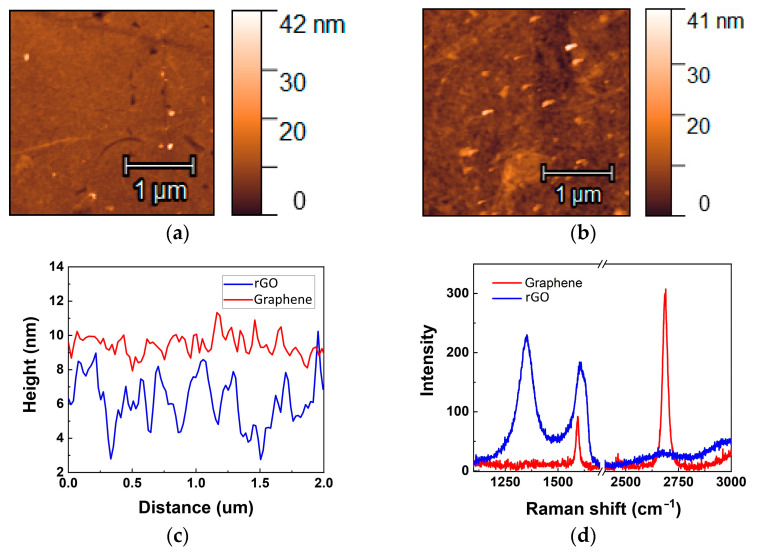
Characterisation of CVD graphene and GO films transferred on IDE electrodes. AFM-image of graphene (**a**) and rGO film (**b**) on glass surface (between electrodes). Height profiles of cross-sections of AFM images, shown on (**a**) and (**b**) for graphene and rGO, respectively (**c**). Raman spectra for graphene and reduced GO films on glass surface (**d**).

Though different approaches were used in this work to deposit CVD graphene (wet-transfer PMMA-assisted method) and GO (spin-coating of preactivated surface), we aimed to achieve a monolayer film for both approaches. The CVD monolayer graphene film was transferred on IDEs, demonstrating an intact surface (Figure 1a). The GO film consists of interconnected individual flakes with an average size of about several microns (Figure 1b). It should be noted that the substrate of commercial IDEs is not atomically flat itself, with high roughness of mechanically polished glass. Therefore, the roughness of the graphene surface is defined mostly by the substrate beneath it and was estimated as 0.46 ± 0.03 nm (Figure 1c). GO, due to its internal flexible structure and the presence of a high number of sp^3^ defects, demonstrates a roughness of 1.3 ± 0.1 nm, which is more than two times higher than for CVD graphene. In addition, CVD graphene provides planarization of the surface, and the GO film provides conformal coverage of the glass surface, which contributes to the roughening of its structure. The peak-to-peak surface height difference is estimated as 2.2 ± 0.5 nm and 5.5 ± 1.3 nm for graphene and GO films, respectively (Figure 1c).

The Raman spectra of transferred CVD graphene show a low defect density of the structure and its high quality (Figure 1d). The ratio of intensities of 2D and G bands confirms the monolayer presence between electrodes. The defect density can be estimated based on the ratio of intensities of G and D bands. However, graphene on glass has low Raman signal intensities and the D band can be measured on the level of noise that proves high quality of graphene (Table 1). On the contrary, rGO demonstrates a high number of defects even after reduction and almost the absence of a 2D band. This confirms that the surface still contains vacancies and other sp^3^ defects that give an impact in the high channel resistance, which is more than 100 times higher than for CVD graphene. The contact resistance between rGO flakes also can affect the conductivity of the channel. Based on the above, it was clear that the higher roughness and presence of higher number of defects in rGO compared to CVD graphene are involved in the different sensitive properties of the two types of devices.

### 3.2. Aptasensors Assembly using PBASE Linker

**Figure 2:**
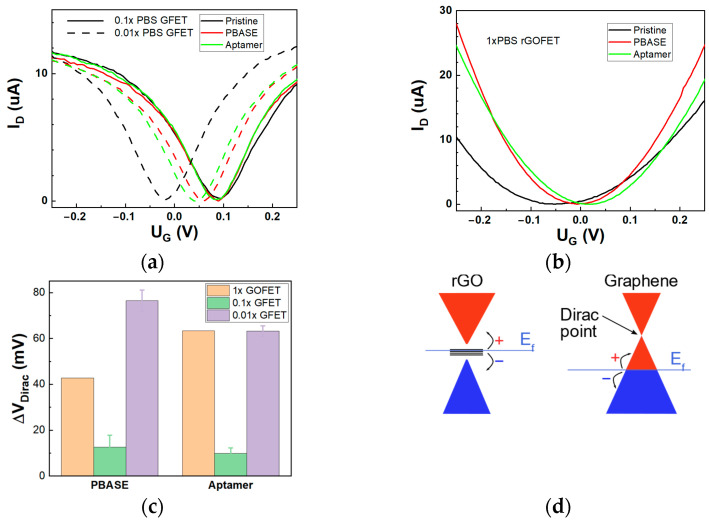
Immobilization of aptamer on the channel of FET. Change in I_D_ – V_G_ curves for GFETs (in 0.1× and 0.01×PBS) (**a**) and rGO-FET (in 1×PBS) (**b**) after assembling to aptasensor (V_DS_ = 100 mV). Dirac point shift change for each step of sensor assembly for GFET and rGO-FET (**c**). Schematic illustrations of electrical response mechanism in GFET and rGO-FET (**d**).

The rGO-FET and GFET chips were assembled into the aptasensors as described in Materials and Methods and were used to analyze the electrical response to NT-proBNP presence in 0.01×PBS—buffer solution. Measurements in 0.01×PBS were previously shown to demonstrate high sensitivity to dissolved analyte molecules [10]. We observed some differences in characteristics of GFET and rGO-FET during the assembling process (Figure 2a,b). PBASE attachment notably affects the electrical properties of both devices leading to p-type doping by the NHS groups [22]. The transconductance was estimated based on the slope of the linear part of p-type branch of I_D_ − V_G_ curves, and the value was similar for both types of bare devices. Transconductance for the devices with the same form factor is defined mainly by the charge carriers mobility and the capacity [23]. We assume that charge carriers’ mobility should be lower for graphene oxide due to the presence of defects, but higher surface area can increase the GO capacity, resulting in almost equal initial transconductance. Transconductance change Δg_m_ after aptasensor assembly was calculated as Δg_m_ = g_m_ − g_m(bare)_, where g_m(bare)_ is the transconductance of the bare FET (Table 1). We observed that in case of GFET, g_m_ was increased only by 10%, while for the rGO-FET configuration, the increase in values was more than 80%. The transconductance increase can be associated with ionic redistribution in the Stern layer that can modulate the thickness of the EDL by planarization of rGO surface [24], thus leading to higher sensitivity of an initially rough surface to the change of absorption layer thickness [25]. The Dirac point shift ΔV_Dirac_ was calculated as ΔV_Dirac_= V_Dirac_ − V_Dirac(bare)_, where V_Dirac_—measured Dirac point and V_Dirac(bare)_—Dirac point of the bare rGO-FET or GFET, and is shown at Figure 2c. We observed that for high ionic strength (small EDL thickness), the Dirac point shift is almost negligible for the CVD graphene, and only for diluted PBS solution can the reliable signal be observed (Figure 2c). At the same time, the sensitivity of rGO-FET is high enough in 1×PBS solution. We assume this difference in sensitivity is due to effects of electrostatic gating from the aptamer to the semimetal graphene [3,26] and semiconducting monolayer of rGO [4] (Figure 2d). When graphene is highly doped (p-type, in our case), most of the states are occupied and the relative increase in the total number of charge carriers is minor. The higher sensitivity in graphene can be observed when the Dirac point is close to the state with minimal charge carriers’ concentration at low ionic strength solution due to the semimetal nature of graphene. For rGO, the trapped states in the bandgap are more sensitive to the electrostatic gating, which can provide higher sensitivity but with a higher level of noise.

Noticeable difference is observed after aptamer binding to PBASE, resulting in the opposite shift of the Dirac point for rGO and graphene FETs. In the case of GFET, we observed a left shift, while for rGO-FET, we observed a right shift of the Dirac point, which is consistent with p-type doping of graphene by negative charges from phosphate groups [10]. However, in the case of GFET biosensors, previous results from the literature are not consistent, demonstrating both negative and positive shifts in Dirac points after aptamer immobilization [27,28]. Herein, we suggest that a competitive effect of electrostatic gating and charge transfer from the aptamer can play a critical role in the direction of the Dirac point shift. In that way, the initial doping of graphene can define the aptasensors’ response. In our research, the graphene is heavily p-type doped when measured in 0.01×PBS and weakly n-type doped in 0.1×PBS (Figure 2a). Thus, when the aptamer brings additional negative charges, there is almost no response in solution with high ionic strength. For lower ionic strength, the concentration of charges near the Dirac cone can be easily modulated, thus increasing the sensitivity of the GFET channel. For rGO, the bandgap plays the main role in conductivity, able to modulate both the Dirac point and the mobility of charge carriers due to the availability of traps. Moreover, π-π stacking of the aptamer backbone on the graphene basal surface can be responsible for higher doping. The flat nature of CVD graphene facilitates the full coverage of its surface with PBASE. Due to this high-density coverage on the surface of graphene, upon functionalization of PBASE with the aptamer, the capabilities for direct interaction between the aptamer and graphene surface are low. The high-density packaging of aptamers on the graphene surface can lead to redistribution of ions in the Stern layer, thereby decreasing the doping in solutions with low ionic strength, which is then observed as a weak left shift of the Dirac point (Figure 2c). On the other hand, the roughness of rGO can prevent the full surface coverage by PBASE, which allows free spaces for the direct interaction between aptamer and carbon lattice, which brings the charges close to the surface and allows direct charge transfer, thus increasing the hole doping of rGO [29]. This is confirmed by a higher increase of transconductance for rGO-FET during the assembly (Table 1).

### 3.3. NT-proBNP Measurements by GFET and rGO-FET Aptasensors

**Figure 3:**
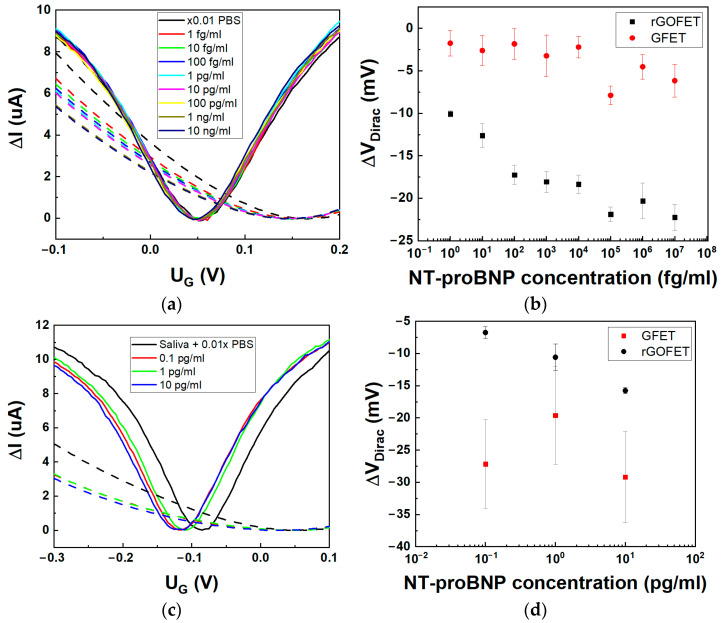
The GFET and rGO-FET aptasensors’ response to NT-proBNP in 0.01×PBS (pH 7.4). (**a**) Transfer curves after stabilization for varied NT-proBNP concentration with no washing steps for GFET (solid lines) and rGO-FET (dashed lines), with applied V_DS_ = 100 mV and 10 mV for rGO-FET and GFET, respectively. (**b**) Dirac point shift for each concentration for GFET (●) and rGO-FET (■, data is reproduced with permission from [10]). (**c**) Transfer curves for three NT-proBNP concentration in AS, dissolved in 0.01×PBS for GFET (solid lines) and rGO-FET (dashed lines), with applied V_DS_ = 50 mV. (**d**) Dirac point shift for each concentration of NT-proBNP in AS, diluted in 0.01×PBS for GFET (■) and rGO-FET (●).

Measurement of NT-proBNP was carried out in 0.01×PBS with Tween to increase the Debye length [30]. Measurements of current-voltage characteristics for stepwise increase of NT-proBNP concentrations (Figure 3a) revealed different Dirac point shifts in terms of the value but similar direction for both GFET- and rGO-FET-based aptasensors (Figure 3b). A noticeable difference is observed for the hole branch of CVCs of these devices. Transconductance did not change for the GFET aptasensor, which corresponds to the previous results on photochemically immobilized GFET with an aptamer [28]. The absence of an effect on the mobility of charge carriers in CVD graphene confirms its intact structure and the absence of trapped states associated with defects. As we observed before for rGO-FET [10], both transconductance and Dirac point shift are more pronounced due to the effect of NT-proBNP binding with the aptamer on both electrostatic doping and direct doping to the trapped states in rGO. The Dirac point shift for CVD graphene is weak and only observed starting from 10 fg/mL, with a fast saturation of the signal for higher concentrations of NT-proBNP (Figure 3b).

To discuss in more detail the difference between GFET and rGO-FET response, we should consider (i) decreasing the aptamer effect on the FET channel when binding with the target and (ii) bringing the net charge from the target that directly affect the graphene channel. In specific circumstances, e.g., varied ionic strength, these effects can compensate each other; hence, the reliability of the biosensor decreases. Firstly, in both cases when the NT-proBNP binds to the aptamer, the electrostatic gating or charge transfer from the aptamer is decreased, which is observed as an opposite (left) shift of the Dirac point. This effect was previously clearly proven using small molecules as analytes that do not carry substantial charge [5]. This effect is also observed for an rGO biosensor where the maximum amplitude of the Dirac point shift is 20 mV, which corresponds to the initial shift after aptamer immobilization. As we discussed previously, aptamers are not interacting with graphene in GFET due to high PBASE coverage density and are rather free-standing. Isoelectric point of NT-proBNP is 8.5 [31], and hence, it is positively charged in PBS at neutral and lower pH. Upon binding to GFET, both negative charges from the phosphate groups of the aptamer and positive charges from NT-proBNP are simultaneously brought close to the graphene surface. In the case of the rGO-FET sensor, the aptamer is already close to the surface prior to binding and significant change is observed with binding of the positively charged NT-proBNP. In conclusion, the response signal from the CVD graphene biosensor is smaller and the noise level is higher in comparison to the rGO-FET biosensor, as shown in Table 2.

The selectivity test was performed for both described types of aptasensors after long-term storage in normal conditions, using biomarkers cTnI and proBNP (Figure S1). Good selectivity (low sensitivity) was demonstrated for both types of biosensors towards cTnI, which corresponds to the results demonstrated before [10]. Unexpectedly, higher sensitivity was demonstrated towards the proBNP peptide. NT-proBNP is a part of prohormone proBNP; however, aptasensors generally do not demonstrate sensitivity towards proBNP [32]. We assume that specific stacking of the aptamer on the graphene surface can increase the sensitivity to proBNP. Moreover, clear concentration dependence in sensitivity was observed for GFET, which can support the scheme of larger distance of charges brought by aptamer from the flat CVD graphene surface. On the other hand, we can expect some degradation of the sensing properties for both types of aptasensors after long-term storage.

One of the potential applications of aptasensors targeting NT-proBNP biomarker is the possibility of non-invasive biomarker control in saliva. To this end, we tested the developed aptasensors for detection of NT-proBNP in spiked AS. Three concentrations were prepared: 0.1 pg/mL, 1 pg/mL, and 10 pg/mL in AS dissolved in 0.01×PBS, which corresponds to the working range of the biomarker in real saliva samples from patients with HF [33].

A comparison between the GFET and rGO-FET aptasensors response is given in Figure 3c. Interestingly, we observe a similar shift for both I_D_-V_G_ curves of graphene and rGO FETs in pure AS solution due to a change in ionic strength and pH brought by AS. Saliva has lower pH compared with PBS, and the Dirac point shifts left in accordance with graphene sensitivity [34]. When replacing PBS with dissolved artificial saliva, the Dirac point shifts from 55 ± 1 mV to –83 ± 4 mV and from 176 ± 7 mV to 51 ± 4 mV for GFET and rGO-FET, respectively. As with PBS solution, we observe a higher dependence of both Dirac point shifts and transconductance change for rGO-FET aptasensors with increasing concentration of NT-proBNP (Figure 3d). Moreover, the signal sensitivity in the rGO-FET biosensor was increased from 2.5 mV/dec in 0.01×PBS to 5 mV/dec in AS solution. On the other hand, the response of GFET was not stable due to the heavy n-type doping in AS solution and the increase in the noise level. Based on the above, the GO-based aptasensors are evidently more effective in sensing small peptides like NT-proBNP in saliva. However, their accuracy is hampered due to the presence of both the bandgap and traps, which influence the sensitivity of the device and can affect biosensor reproducibility. The preparation steps can influence the final performance of the devices. We have compared the possible advantages (+) and disadvantages (−) of each step of aptasensor construction in Table 3.

## 4. Conclusions

We have developed a CVD graphene-based FET biosensor with non-covalently linked aptamer for NT-proBNP detection and compared its performance with an rGO-based FET biosensor. The results indicate that when choosing the proper technology for biosensors development, one should consider not only the internal properties of the sensing materials (like graphene vs. graphene oxide) but also the analyte and sensing mechanism as well. The NT-proBNP molecule is a small peptide that can interact with aptamers in different manners, which can result in large errors in measurement. We demonstrated that the aptamer-based assay for NT-proBNP analysis can be performed in diluted artificial saliva. To increase the sensitivity (decrease the noise), materials with a larger bandgap and presence of trapped states, such as rGO-FET, are preferred for construction of point-of-care devices.

In this paper, we only limit the comparison to the rGO and graphene-based FETs with IDE electrodes for sensing small peptides such as NT-proBNP based on its conjugation with specific aptamer. Additional research is needed on comparison of stability and repeatability of both types of biosensors in long-term use. For example, the properties of CVD graphene-based sensors are particularly suitable for real-life application, e.g., as graphene tattoo-based biosensors. This novel technology could enable formation of two-and three-layer structures with CVD graphene, with the possibilities for additional chemical or structural modification to increase the sensitivity of the device.

## Figures and Tables

**Table 1:** The comparison of resistance (R), transconductance (g_m_), its change (∆g_m_) after biosensor assembly, intensity ratio of Raman bands of graphene and rGO channels.

Parameter	GFETs *	rGO-FETs **
R, kOhm	0.026 ± 0.001	2.27 ± 0.05
g_m_, µS	84.7 ± 0.7	85.4 ± 5.3
∆g_m_, µS	7.5 ± 1.6	47.1 ± 4.0
Raman band intensity ratio	≥5.11	0.76

* GFET—GFET channel, N = 2; ** rGO-FET—rGO-FET channel, N = 2.

**Table 2:** Analytical performance of aptasensors based on CVD GFET and rGO-FET.

Parameters	GFETs	rGO-FETs
Dynamic range, pg/mL	10^−2^–10^2^	10^−2^–10^2^
LOD, pg/mL	1	0.1
Sensitivity, mV/dec	~0.7	~2.5

**Table 3:** The preparation steps of aptasensors based on GFET and rGO-FET.

Assembling Step	GFETs	rGO-FETs
Commercial IDE electrodes	Easy fabrication (+) Reproducibility (+) Top contact (−)	
2. Deposition of sensitive layer	Bad reproducibility of graphene positioning (−) Excellent conductivity and intact structure (+) Good surface area (+)	Excellent reproducibility of GO layer (+) Weak conductivity and layer of polycrystalline structure (−) Excellent surface area (+)
3. Annealing	Not necessary (+)	Mandatory (−)
4. Linker immobilization	Good density (+) High coverage (+)	High density (+) Low coverage (−)
5. Aptamer immobilization	Small EDL (−)	Increased EDL (+)

## Data Availability

The data presented in this study are available on request from the corresponding authors.

## Supplementary Materials

The following supporting information can be downloaded at: https://www.mdpi.com/article/10.3390/bios14050215/s1, Figure S1: Sensitivity of rGO-FET and GFET based biosensors to nonspecific biomarkers (cTnI and proBNP).

## Abbreviations

APTES	(3-aminopropyl) triethoxysilane
AS	artificial saliva
BSA	bovine serum albumin
cTnI	cardiac troponin I
CVC	current voltage characteristics
CVD	chemical vapor deposition
DMF	dimethylformamide
EDL	electrical double layer
ETA	Ethanolamine
FET	field effect transistor
GFET	graphene FET
HF	heart failure
GO	graphene oxide
IDE	interdigitated electrodes
IPA	isopropyl alcohol
LOD	limit of detection
NMP	N-methyl-2-pyrrolidone
NT-proBNP	N-terminal proBNP
PBASE	pyrenebutyric acid N-hydroxysuccinimide ester
PBS	phosphate buffer silane
PMMA	poly(methyl methacrylate)
POC	point of care
proBNP	B-type natriuretic peptide
rGO	reduced graphene oxide
