# Supplementary material for: Comparative Study of Field-Effect Transistors Based on Graphene Oxide and CVD Graphene in Highly Sensitive NT-proBNP Aptasensors
Source: Biosensors (Basel). 2024 Apr 26;14(5):215. doi: 10.3390/bios14050215 (PMC11117807; doi:10.3390/bios14050215)
Supplement: Supplementary file 1 [file biosensors-14-00215-s001.zip › biosensors-2936992-supplementary.pdf]

biosensors

MDPI

Supplementary Information

Comparative Study of Field-Effect Transistors Based on Graphene Oxide and CVD Graphene in Highly Sensitive NT-proBNP Aptasensors

Anastasiia Kudriavtseva [1,†], Stefan Jarić [2,†], Nikita Nekrasov [1], Alexey V. Orlov [3], Ivana Gadjanski [2], Ivan Bobrinetskiy [2], Petr I. Nikitin [3,*] and Nikola Knežević [2,*]

[1] Center for Probe Microscopy and Nanotechnology, National Research University of Electronic Technology, Moscow, Zelenograd 124498, Russia;
[2] BioSense Institute—Research and Development Institute for Information Technologies in Biosystems, University of Novi Sad, 21000 Novi Sad, Serbia; sjaric@biosense.rs (S.J.); igadjanski@biosense.rs (I.G.);
[3] Prokhorov General Physics Institute of the Russian Academy of Sciences, Moscow 119991, Russia;
* Correspondence: petr.nikitin@nsc.gpi.ru (P.I.N.); nknezevic@biosense.rs (N.K.)
† These authors contributed equally to this work.

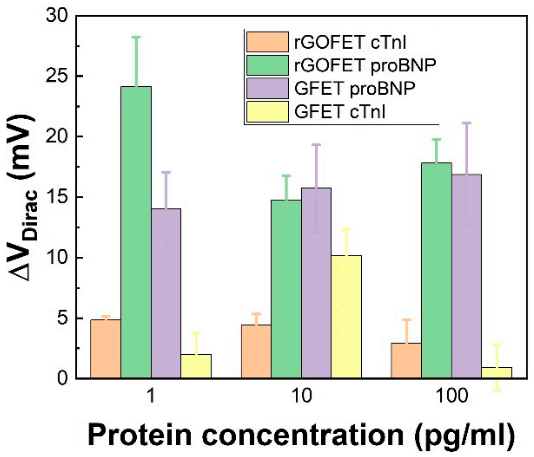

Figure S1. Sensitivity of rGO-FET and GFET based biosensors to nonspecific biomarkers (cTnI and proBNP).
